# Tooth Movement out of the Bony Wall Using Augmented Corticotomy with Nonautogenous Graft Materials for Bone Regeneration

**DOI:** 10.1155/2014/347508

**Published:** 2014-08-27

**Authors:** Kye-Bok Lee, Dong-Yeol Lee, Hyo-Won Ahn, Seong-Hun Kim, Eun-Cheol Kim, Igor Roitman

**Affiliations:** ^1^Department of Orthodontics, School of Dentistry, Kyung Hee University, No.1 Hoegi-dong, Dongdaemun-gu, Seoul 130-701, Republic of Korea; ^2^Department of Oral and Maxillofacial Pathology and Research Center for Tooth & Periodontal Regeneration (MRC), School of Dentistry, Kyung Hee University, Seoul 130-701, Republic of Korea; ^3^Division of Periodontology, Department of Orofacial Science, University of California, San Francisco, CA 94122, USA

## Abstract

This prospective randomized split-mouth study was performed to compare the effects of augmented corticotomy with those of different nonautogenous bone graft materials combined with orthodontic tooth movement in dogs. Decortication was performed on the buccal bone surface of 6 male beagle dogs that were randomly assigned to receive grafts of deproteinized bovine bone mineral, irradiated cortical bone, or synthetic bone. Immediate orthodontic force was applied to the second and third premolars for buccal tipping for 6 weeks. The pocket depth and width of keratinized tissue (WKT) were measured. Histologic and histomorphometric analyses were performed. The probing depth, WKT, and ratio of the area of new bone to that of total bone on the buccal side were not significantly different between groups. All groups had considerable new bone formation on the pressure side. New bone formation on the buccal side and buccal plate formation in the coronal direction along the root surfaces were induced by the bone-derived and PDL-derived mesenchymal matrix, respectively. The angular change between groups was significantly different (*P* < 0.001). Augmented corticotomy using nonautogenous graft materials facilitated tooth movement without fenestrations and accelerated new bone formation on the pressure side.

## 1. Introduction

During orthodontic treatment, tipping movement of teeth commonly occurs. However, buccolingual movement exceeding the alveolar housing may result in bony dehiscence at the crest area and cause gingival recession, especially in the decompensation of class III patients [1], maximum retraction of anterior teeth in bialveolar protrusion patients [2], and transverse expansion of upper molars by rapid palatal expansion [3]. Wainwright et al. [4] reported that once the cortical plate has been penetrated during tipping movement, the buccal root surface becomes devoid of cortical bone. Repair, not regeneration, of the perforation site took place only after the teeth had relapsed. However, no histologic studies have demonstrated the regeneration or reestablishment of the cortical plate [5, 6].

Corticotomy-facilitated tooth movement with alveolar bone augmentation may facilitate the retention of the periodontal ligament, thereby preventing bony dehiscence and gingival recession [7, 8]. Wilcko et al. [9] reported that alveolar augmentation could reinforce the dentoalveolar deficiency when the teeth were tipped labially and suggested that alveolar bone augmentation could provide patients with a more structurally intact periodontium at the completion of the orthodontic treatment. Moreover, corticotomy on alveolar bone resulted in more rapid orthodontic tooth movement than that obtained with conventional methods [10, 11]. The regional acceleratory phenomenon (RAP) can accelerate both hard tissue healing and soft tissue healing by 2- to 10-fold, thus leading to decreased regional bone density [12, 13]. A recent clinical study [7] showed that the augmented corticotomy provided effective decompensation of the mandibular incisors in skeletal class III patients while maintaining labial bone thickness with no periodontal side effects. However, histological observations related to the periodontal reaction to augmented corticotomy are rare.

In recent years, nonautogenous bone grafts, especially synthetic bone substitutes, have played an increasingly crucial role in surgical dentistry. Wilcko et al. [8, 9] reported satisfactory results with a combination of demineralized freeze-dried bone allografts (DFDBA), xenografts, or bioabsorbable alloplastic grafts for alveolar augmentation. They reported only one human histologic case study in which deproteinized bovine bone mineral was used [14]. However, no studies have focused on the influence of graft materials with different origins, especially synthetic bone, when combined with corticotomy and further orthodontic tooth movement. Because most of what is known about the decortication procedures in orthodontic treatment is based on case reports, more laboratory and clinical studies are necessary to better elucidate the biological mechanisms involved at the tissue level. The purpose of this study was to compare the effects of deproteinized bovine bone mineral (DBBM: Bio-Oss; Geistlich Pharma AG, Wolhuser, Switzerland), irradiated cortical bone (ICB: Rocky Mountain Tissue Bank, Aurora, CO), or synthetic bone (SB: MBCP^+^; micromacroporous biphasic calcium phosphate; Biomatlante, Vigneux de Bretagne, France) in an augmented corticotomy procedure for periodontal tissue reconstruction in the context of uncontrolled buccal tipping movement of teeth in beagle dogs.

## 2. Materials and Methods

### 2.1. Animals

This study was a prospective, split-mouth, randomized controlled experimental design using 6 male beagle dogs; dogs were randomly assigned to Groups A, B, and C. Augmented corticotomy was performed with DBBM in Group A, ICB in Group B, and SB in Group C. Groups A and B were used as positive controls in our study because Wilcko et al. [14] had reported them as such, and one of the goals of this study was to evaluate SB in comparison to other options. Buccal tipping of the second (P2) and third premolars (P3) was planned on the upper and lower arches for 6 weeks. All animals were 1-2 years old and weighed between 10 and 13 kg. They were caged individually, fed soft food and a standard diet, and given water ad libitum. The experimental protocols in this study were approved by the Institutional Animal Care and Use Committee (KHMC-IACUC-11-021, KHMC-IACUC-10-070).

### 2.2. Clinical Examination

During the examinations and surgical procedures, the dogs were anesthetized with a mixture of tiletamine-zolazepam (5–10 mg/kg; Zoletil 100; Virbac, Carros, France) and xylazine (5 mg/kg,; Rompun; Bayer Korea Ltd., Seoul, Korea) intramuscularly (IM) and intravenously (IV). Intravenous medications were administered using a catheter in the vessel of the ear. The PD and WKT were measured at the mesial, middle, and distal buccal sides of each tooth with a periodontal probe, and the periodontal conditions at baseline and 6 weeks after the procedure were compared. Following these measurements, scaling was performed. An alginate impression was taken to make a study model to fabricate orthodontic appliances (band and wire). The canine (C) and fourth premolar (P4) were used as anchorage, and thick wire (ø 0.9 mm) with traction hooks was welded to the buccal surface of the bands [15]. At the second (P2) and third premolars (P3), the lingual button was welded on the lingual surface of the band. Immediately after augmented corticotomy, an orthodontic force (200 g) for buccal tipping was applied to the lingual buttons of P2 and P3 for 6 weeks. The period of 6 weeks was considered to be sufficient to form new bone and induce tooth movement in augmented corticotomy.

### 2.3. Surgical Procedure

**Figure 1:**
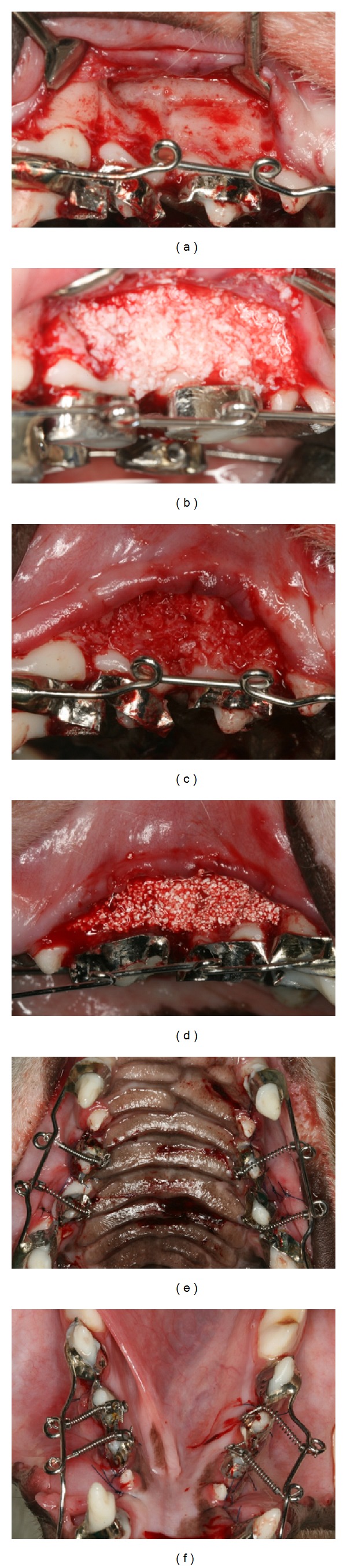
Surgical procedure. Full-thickness flap elevation and corticotomy were performed on the buccal side (a). Deproteinized bovine bone mineral (DBBM) particles (b), irradiated cortical bone (ICB) particles (c), and micromacroporous biphasic calcium phosphate (SB: MBCP^+^) particles (d) were grafted. Primary closure was obtained, and activation of P2 and P3 for buccal tipping was started with a closed coil spring (200 g) ((e) and (f)).

After 2 weeks, the animals were anesthetized to fix the orthodontic appliances to the teeth, and lidocaine hydrochloride (2% lidocaine with 1 : 100,000 epinephrine; Huons Co., Seoul, Republic of Korea) was infiltrated into the surgical sites for local anesthesia. An intrasulcular incision was made from the canine to the first molar. Then, a full-thickness flap was elevated carefully. The circumscribing corticotomy was performed at the buccal bone surface only with a round bur (ø 1.5 mm) under sterile saline irrigation. The DBBM, ICB, and SB were grafted (1 cc per surgical site) onto the buccal bone surface randomly. Each animal received only one graft depending on the group it was assigned to. The mucoperiosteal flaps were repositioned and sutured with 5–0 nylon. Primary closure was obtained. A closed coil spring with a tensile strength of 200 g was applied in the buccolingual direction immediately, and P2 and P3 were activated for buccal tipping (Figure 1).

After surgery, gentamicin (Dongwha-Pharm. Co., Seoul, Korea) and ketoprofen (Ketopro; Uni Biotech, Chungnam, Korea) were administered IM two times daily for 6 days, and irrigation with 1% chlorhexidine-gluconate solution was performed simultaneously for infection control. Scaling and mechanical plaque control were performed once per week. Six weeks after the procedure, PD and WKT were measured under general anesthesia, and the animals were humanely euthanized with an overdose of thiopental sodium.

### 2.4. Histological Examination

The mandibles and maxillae of all experimental animals were dissected, and block specimens were obtained. The retrieved block specimens were rinsed in sterile saline and immediately immersed in 10% neutral buffered formalin for 14 days. Decalcification was performed using 5% nitric acid for 6 days [16]. Following decalcification, the specimens were dehydrated through a series of ethanol and embedded in paraffin. Buccolingual sections were sliced with the microtome set at 5 *μ*m and stained with Masson's trichrome [11, 17].

**Figure 2:**
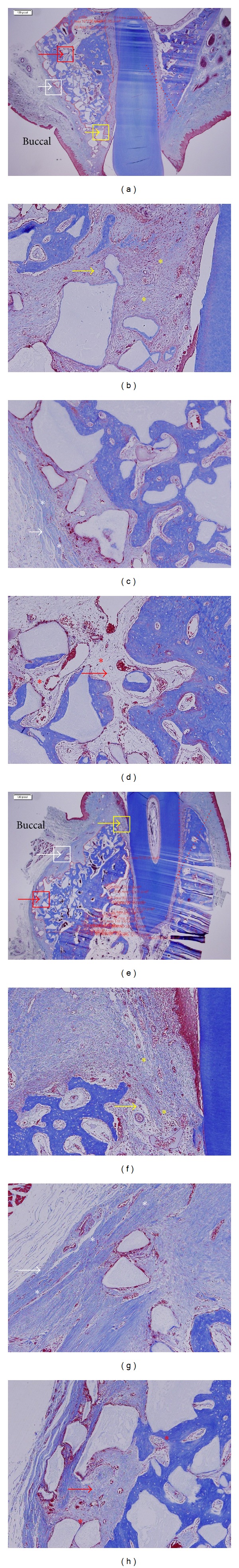
Methods of measurements. (a)–(d) Maxilla, buccal tipping angle (red dotted line) and buccal bone wall area. (e)–(h) Mandible, buccal tipping angle (red dotted line) and buccal bone wall area. Yellow arrow indicates PDL-derived mesenchymal matrix, white arrow indicates buccal mesenchymal matrix, and red arrow indicates bone-derived mesenchymal matrix. (b), (f) Yellow arrow and stars indicate PDL-derived mesenchymal matrix. New bone formation was observed around grafted particles that were embedded in PDL-derived mesenchymal matrix. (c), (g) White arrow and stars indicate the buccal mesenchymal matrix, which plays the role of thick periosteum. (d), (h) Red arrow and stars indicate bone-derived mesenchymal matrix that appears to be loose connective tissue. Masson's trichrome stain. Original magnification for (a) and (e): ×12.5; for (b), (c), (d), (f), (g), (h): ×100.

Histologic evaluations were performed using a light microscope (Olympus BX51; Olympus, Tokyo, Japan) equipped with an Olympus DP21 microscope camera. CellSens imaging software (version 1.6; Olympus Corporation, Tokyo, Japan) was used to measure the angle and bone area. After microscopic examination, a photograph of each slide was taken, and the resulting images were saved. The tipping angle and buccal bone area were measured independently by 3 examiners who were blinded to all group information. The buccal tipping angle was measured from the reversal line of the palatal/lingual bone to the lingual/palatal sides of the root surface. The total buccal bone wall area and old bone area were measured, and the new bone area was calculated by subtracting the old bone area from the total bone area. Grafted particles embedded in and bridged with newly formed bone were included in the new bone area, but floating particles without new bone in soft connective tissue were excluded (Figure 2).

### 2.5. Statistical Analysis

Descriptive statistics (mean ± SD) for each parameter were evaluated in all groups. Within each group, the measurements from the mandible and maxilla were not different; therefore, they (the maxilla and mandible) were combined for analysis. The first, second, and third measurements were compared within each group using Pearson's correlation coefficient, which was higher than 0.98 at the 95% confidence level; therefore, the mean value of the three datasets was used for further description. For histomorphometric analysis, the buccal tipping angle and ratio of new bone area/total bone area were analyzed by the nonparametric Kruskal-Wallis test because the measured number of teeth in each group was not normally distributed. The paired *t*-test was used to compare the soft tissue measurements at baseline and 6 weeks after the surgery. Statistical significance was defined at* P* < 0.05. Three examiners measured angles and bone area. Intraclass correlation analysis was used to analyze interexaminer difference. All statistical analyses were performed using SPSS statistical software (version 18.0; SPSS Inc., Chicago, IL).

## 3. Results 

All sites showed uneventful healing, with no to minimal signs of inflammation.

### 3.1. Soft Tissue

Significant differences were observed between the groups for PD but not WKT. The PD at baseline and at 6 weeks after surgery (1.37 ± 0.59 mm and 1.99 ± 0.91 mm, resp.) was significantly different (*P* < 0.001); however, the difference was only approximately 0.4 mm, and this difference was regarded as not physiologically or clinically significant. The WKT at 6 weeks after surgery (3.94 ± 1.04 mm) did not differ significantly from that at baseline (3.90 ± 1.15 mm) (*P* = 0.704). These results were combined from all of the groups.

### 3.2. Buccal Tipping Angle

**Figure 3:**
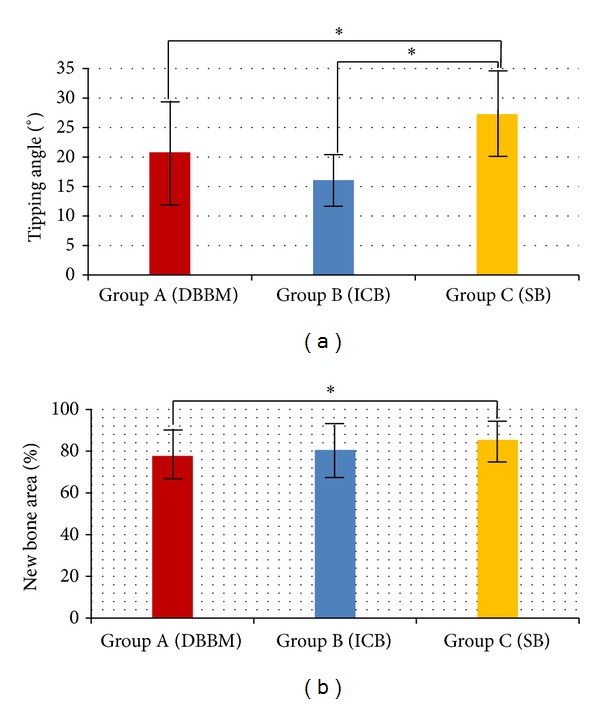
(a) Buccal tipping angle (°). (b) New bone area (NB, %). *P < 0.05.

All groups had uncontrolled buccal tipping of P2 and P3. There was a significant difference in the angular change between groups (Group A, 20.81 ± 8.07°; Group B, 16.08 ± 4.14°; and Group C, 27.26 ± 7.27°) (Figure 3(a)). Statistical significance was found between groups, specifically between Groups A and B, *P* = 0.137; Groups B and C, *P* = 0.000; and Groups A and C, *P* = 0.019. The intraclass correlation coefficient (*r*) was 0.997 and the correlation was statistically significant (*P* = 0.000).

### 3.3. Ratio of New Bone Area on Pressure Sides

All groups showed significant new bone formation at the buccal sides. The ratio of new bone area to total bone area on the buccal side in the different groups was as follows: Group A, 77.7 ± 11.5%; Group B, 80.6 ± 12.7%; and Group C, 85.4 ± 8.8%. The difference between Groups A and C was statistically significant (*P* = 0.046) (Figure 3(b)). The intraclass correlation coefficient (*r*) was 0.709, and the correlation was statistically significant (*P* = 0.000).

### 3.4. Histological Observations

**Figure 4:**
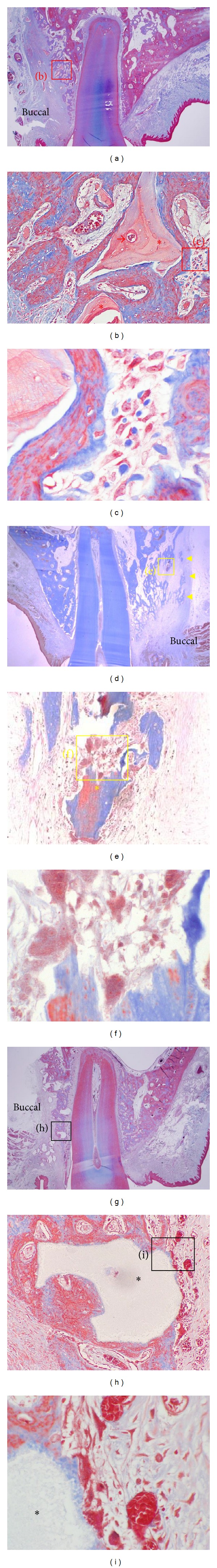
Microphotograph of a buccopalatal section from the maxilla. (a) Deproteinized bovine bone mineral (DBBM) graft. (b) Higher magnification of (a). Red stars indicate DBBM particles that were embedded in and bridged with newly formed bone within the bone-derived mesenchymal matrix. (c) Higher magnification of (b). Active aggregated osteoblasts were observed to form new bone matrix. (d) Irradiated cortical bone (ICB) graft. Most of the grafted ICB particles were resorbed. Grafted ICB particles were embedded in bone-derived mesenchymal matrix, encircled by a newly formed bone wall (yellow arrows). Newly formed bone walls in the mesenchymal matrix appeared to represent buccal bone expansion or bursting. (e) Higher magnification of (d). Grafted particles were bridged with newly formed bone. (f) Higher magnification of (e). Active osteoclasts and osteoblasts are shown. Grafted ICB particles were still resorbed by osteoclasts within the bone-derived mesenchymal matrix. (g) Micromacroporous biphasic calcium phosphate (SB; MBCP^+^) graft. Some of the grafted SB particles were embedded in the newly formed buccal bone wall and faced the PDL-derived mesenchymal matrix. (h) Higher magnification of (g). Grafted SB particles were resorbed by osteoclasts in the PDL-derived mesenchymal matrix and embedded in newly formed buccal bone. Small capillaries were abundant around the grafted particles (black star). (i) Higher magnification of (h). The surface of grafted SB particles was covered with newly formed bone and resorbed by osteoclasts in the process of remodeling. Masson's trichrome stain. Original magnification for (a), (d), and (g): ×12.5; for (b), (e), and (h): ×100; for (c), (f), and (i): ×400.

**Figure 5:**
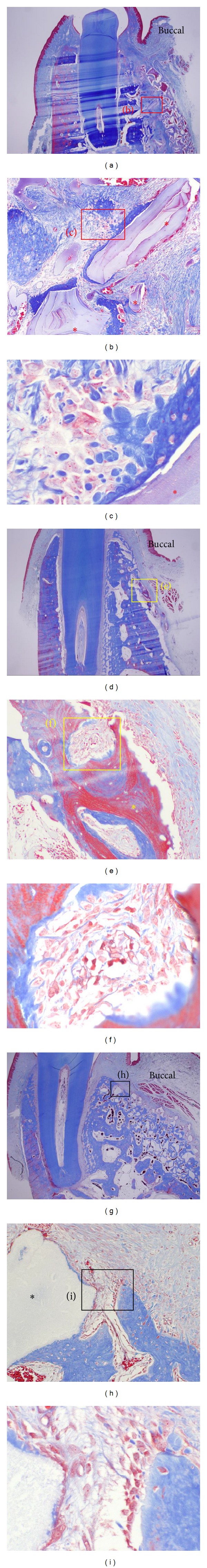
Photomicrograph of a buccolingual section from the mandible. (a) Deproteinized bovine bone mineral (DBBM) graft. (b) Higher magnification of (a). DBBM particles (red star) were embedded in and bridged with newly formed bone within the bone-derived mesenchymal matrix. Resorption by osteoclasts at the buccal side and new bone formation at the inner side by osteoblasts were observed simultaneously. (c) Higher magnification of (b). New bone formation on the surface of the grafted DBBM particles (red star) and active osteoblasts were observed. (d) Irradiated cortical bone (ICB) graft. Most grafted ICB particles were resorbed. Grafted ICB particles were embedded in bone-derived mesenchymal matrix, encircled by a newly formed bone wall. (e) Higher magnification of (d). Grafted ICB particles (yellow star) were still resorbed by osteoclasts within the bone-derived mesenchymal matrix, and newly formed bone was bridged with and formed in the grafted particle. (f) Higher magnification of (e). Active osteoblasts forming new bone were observed inside the grafted ICB particles. (g) Micromacroporous biphasic calcium phosphate (SB; MBCP^+^) graft. Most of the grafted SB particles were embedded in the newly formed bone-derived mesenchymal matrix that formed buccal bone. The thickness of the newly formed bone wall was outstanding. (h) Higher magnification of (g). Grafted SB particles (black star) were embedded in and bridged with newly formed buccal bone. (i) Higher magnification of (h). Active new bone-forming osteoblasts were observed at the outer surface of the buccal bone wall. Masson's trichrome stain. Original magnification for (a), (d), and (g): ×12.5; for (b), (e), and (h): ×100; for (c), (f), and (i); ×400.

In all groups, considerable new bone formation was observed on the pressure side after 6 weeks (Figures 4 and 5).

For histologic evaluation, in this study, the mesenchymal matrix was designated as PDL-derived, bone-derived, or buccal mesenchymal matrix depending on its origin in the PDL, buccal bone, or the thick, dense connective tissue that covered the buccal bone surface and appeared to play the role of periosteum, respectively. The grafted DBBM, ICB, and MBCP^+^ particles were partially or mostly resorbed, embedded in or bridged with new bone, and/or encapsulated by dense fibrous connective tissue. Most grafted ICB particles were resorbed, whereas more MBCP^+^ and Bio-Oss particles remained in a partially resorbed state. The buccal and lingual/palatal crest level was maintained in all groups. On the pressure sides, the apical root surface resorption was localized in the cementum at the lingual/palatal side because of the force of the uncontrolled buccal tipping. The modeling and remodeling pattern of the alveolar bone on the buccal side had distinguishing features irrespective of the graft material.

In the bone modeling/remodeling at the buccal side, two characteristic appearances were observed: one was a large amount of new bone formation on the buccal side induced by the bone-derived mesenchymal matrix, and the other was new bone formation induced by the PDL-derived mesenchymal matrix producing a buccal bone plate in the coronal direction along the root surfaces. Bone modeling/remodeling was not localized and was observed in all alveolar and basal bone buccolingually. Dense buccal mesenchymal matrix covered the buccal bone surfaces and appeared to protect the bone area.

## 4. Discussion

The augmented corticotomy procedure has expanded the frontier of conventional orthodontic treatment. Generally, the labial and lingual cortical plates at the level of the incisor apex are considered to be the anatomic limits of tooth movement [18]. Although a basic axiom in orthodontics is that “bone traces tooth movement,” the ratio of bone modeling to tooth movement varies according to the direction of orthodontic tooth movement. Buccolingual tipping exceeding alveolar housing may result in bony dehiscence at the crest area and gingival recession, especially in the thin biotype. During presurgical decompensation in class III patients, labial tipping of the lower incisors up to 8–10° is generally expected [19]. In our study, although the amount of labial tipping exceeded twice that of the clinical outcome, the periodontal tissue was surprisingly well maintained at the crest level on the pressure sides. The extent of buccal tipping indicates that the native buccal bone plate had been totally resorbed and was replaced by a newly formed buccal bone plate. Corticotomy-facilitated tooth movement with alveolar bone augmentation is likely to facilitate the retention of the periodontal ligament, thus preventing bony dehiscence and gingival recession [7, 8, 20].

The concept of corticotomy relies on creating bony blocks with embedded teeth that can be moved rapidly with strong forces [21–23]. However, Wilcko et al. [8] suggested that transient localized demineralization/remineralization occurs after corticotomy and that the demineralization of the alveolar bone over the root surfaces leaves the collagenous soft tissue matrix of the bone, which can be carried with the root surface and then remineralized following the completion of the orthodontic treatment. They named this process “bone matrix transportation.” However, in our study, these phenomena were observed as bone modeling/remodeling. In this study, we observed the following two characteristic appearances in the bone modeling/remodeling on the buccal side: (1) a considerable amount of new bone was formed by the bone-derived mesenchymal matrix and (2) new bone formation in the coronal direction along the root surface was induced by the PDL-derived mesenchymal matrix. Bone modeling/remodeling was not restricted to the local area; rather, it was observed buccolingually throughout the alveolar and basal bone. This bone metabolism is considered to be a part of the simultaneously coordinated modeling and remodeling induced by the different types of mesenchymal matrix and not representative of the sequential process of bone matrix transportation as put forth by Wilcko et al.

Augmented corticotomy with a bone graft is a complex procedure that considers multiple factors, such as the timing of orthodontic force application, design and extent of corticotomy, and biological characteristics of the bone graft material. As the length of the RAP is known to be approximately 4 months [24], orthodontic tooth movement should be initiated as early as possible. A previous study regarding orthodontic tooth movement into grafted sites showed a slight increase in the rate of tooth movement immediately after grafting when compared to nongrafted control sites [25]. In our clinic, augmented corticotomy has been performed immediately before the target tooth movement in the same manner used in the present experimental design. The original corticotomy-facilitated orthodontic treatments involved buccal and lingual osteotomy cuts with orthopedic forces, and the use of alveolar augmentation with a demineralized bone graft was advocated to cover any fenestrations or dehiscences and to increase the bony support for both the teeth and the overlying soft tissues [8, 9]. Some case reports showed that selective corticotomy limited to the buccal and labial surfaces reduced the operation time and postoperative patient discomfort [26, 27]. However, in our study, only buccal corticotomy was designed to cover the target area of the thin buccal plate on the pressure sides.

An ideal bone graft substitute should have osteoconductive, osteoinductive, and osteogenic properties. Autogenous bone grafts are the gold standard among the graft materials because they provide all of these properties and contain viable cells that can proliferate and contribute to the formation of new bone [28]. However, they have several limitations, such as the perioperative pain and morbidity associated with harvesting the graft, uncertain quality and quantity of the graft, and limited graft shapes and sizes. To overcome these limitations, nonautogenous bone grafts are being utilized more commonly, and the current study was conducted to investigate the effect of various nonautogenous bone grafts, including allografts, xenografts, and alloplasts, on augmented corticotomy. The DBBM used in our study (Bio-Oss) was selected as a positive control because it is one of the most widely used xenograft materials and is hydroxyapatite of bovine origin [29], and it has well-established satisfactory outcomes in periodontal regeneration [30]. The structural properties of Bio-Oss, such as high porosity and the presence of hydroxyapatite crystals, provide sufficient surface area and stability for the migration and adhesion of osteogenic cells. For mineralized bone allografts, irradiated cancellous bone (ICB) obtained from human cadaver sources is often used [31]. Sterilization by irradiation is performed to reduce the incidence of infection [32]. In the present study, cortical ICB was used because it is resorbed more slowly than the cancellous type. For synthetic alloplasts, a hydroxyapatite/tricalcium phosphate (HA/TCP) mixture is the first choice [33]. Hydroxyapatite provides long-term stability, and *β*-TCP releases ions that form acellular apatite crystals. In the current study, MBCP^+^ (a 20:80 HA:*β*-TCP mixture) was used because it was reported to have a bone formation rate 20% higher than that of conventional MBCP (a 60:40 HA:*β*-TCP mixture).

Surprisingly, all groups had a buccal plate that was newly formed at the buccal side from the crestal area to the apical area of the pressure side. In this study, the SB group had the largest amount of new bone formation, despite the large angular changes during orthodontic tooth movement. These results suggest that the osteoconductive synthetic graft material is not inferior to other grafts when used for augmented corticotomy. Although statistically significant differences were observed, these differences are not considered to be physiologically or clinically significant. All of the graft materials contributed to new buccal bone formation. The synthetic bone substitute is advantageous because it is inexpensive, does not elicit an immune reaction, and is available in sufficient quantities; therefore, it is expected to be the preferred option in augmented corticotomy.

This pilot study had certain limitations regarding the experimental design. Further studies with a larger sample size and negative control (corticotomy only) are necessary. Additionally, the 1-walled defect model, which would be the most challenging model for securing of graft materials, differs between dogs and humans. The buccal alveolar wall is more concave in dogs than in humans; therefore, these results should be applied to clinical situations cautiously.

## 5. Conclusion

Augmented corticotomy effectively reestablished the periodontal soft and hard tissue after buccal tooth tipping movement on the pressure sides, regardless of the type of graft material used. The synthetic bone graft group, which had the largest angular changes during tipping, had the most new bone formation and maintained the soft tissue intact. This technique may be beneficial for preventing loss of the periodontal support during orthodontic tooth movement beyond the thin alveolar bone plate.
